# Respiratory viruses in patients with influenza-like illness in Senegal: Focus on human respiratory adenoviruses

**DOI:** 10.1371/journal.pone.0174287

**Published:** 2017-03-22

**Authors:** Mbayame Ndiaye Niang, Ndeye Sokhna Diop, Amary Fall, Davy E. Kiori, Fatoumata Diene Sarr, Sara Sy, Déborah Goudiaby, Mamadou Aliou Barry, Malick Fall, Ndongo Dia

**Affiliations:** 1 Institut Pasteur de Dakar, Unité de Virologie Médicale, Dakar, Sénégal; 2 Institut Pasteur de Dakar, Unité d’Epidémiologie des maladies infectieuses, Dakar, Sénégal; 3 Département de Biologie Animale Faculté des Sciences et Techniques Université Cheikh Anta DIOP de Dakar, Dakar, Senegal; Kliniken der Stadt Köln gGmbH, GERMANY

## Abstract

**Background:**

Human adenoviruses (HAdVs) are highly contagious pathogens that are associated with a wide spectrum of human illnesses involving the respiratory tract. In the present study, we investigate the epidemiologic and viral molecular features of HAdVs circulating in Senegal after 4 consecutive years of sentinel surveillance of influenza-like Illness cases.

**Methodology and results:**

From January 2012 to December 2015 swabs were collected from consenting ILI outpatients. Adenoviral detection is performed by rRT-PCR with the Anyplex^™^ II RV16 Detection kit (Seegene) and molecular characterization was performed using a partial hexon gene sequence. 6381 samples were collected. More than half of patients (51.7%; 3297/6381) were children of ≤ 5 years. 1967 (30.8%) were positive for HAdV with 1561 (79.4%) found in co-infection with at least one another respiratory virus. The most common co-detections were with influenza viruses (53.1%; 1045/1967), rhinoviruses (30%; 591/1967), enteroviruses (18.5%; 364/1967) and RSV (13.5%; 266/1967). Children under 5 were the most infected group (62.2%; 1224/1967; p <0.05). We noted that HAdV was detected throughout the year at a high level with detection peaks of different amplitudes without any clear seasonality. Phylogenetic analysis revealed species HAdV-C in majority, species HAdV-B and one HAdV- 4 genome type. The 9 HAdV-B species like strains from Senegal grouped with genome types HAdV-7, HAdV-55 and HAdV-11 as shown by a phylogenetic branch with a high bootstrap value of (88%).

**Conclusion:**

In conclusion, the results of the present study suggest strong year-round HAdV activity in Senegal, especially in children up to 5 years of age. Molecular studies revealed that the dominant species in circulation in patients with ILI appears to be HAdV-C and HAdV-B species. The circulation of though HAdV-7 and HAdV-55 genome types is of note as these serotypes are recognized causes of more severe and even fatal acute respiratory infections.

## Background

Human adenoviruses (HAdVs) are highly contagious pathogens that are associated with a wide spectrum of human illnesses involving the respiratory, ocular, gastrointestinal, and genitourinary systems [1]. They belong to the family Adenoviridae, genus Mastadenovirus with seven species (A-G), including each various types [2]. Ubiquitous in the environment, HAdVs are non-enveloped, double stranded DNA viruses that vary in size from 70 to 100 nm [3]. HAdVs are recognized as a common cause of respiratory infection in persons of all ages. The illnesses range from influenza-like fever and discomfort to pneumonia and death [4]. Indeed, HAdVs infections are usely mild but some groups such as very young children, elderly, immunocompromised persons, or persons with underlying pulmonary or cardiac disease, might be at higher risk degree for severe disease [5,6,7,8]. The most common HAdVs species that cause respiratory tract infections in children are B (HAdV-B3 and B7) and C (HAdV-C1, C2, and C5). Serotypes B3, B7, and B21 are the most frequent strains responsible for epidemics of acute febrile respiratory disease [9]. Circulating HAdVs can vary temporally and geographically with possibility of emergent genomic variants which can be associated with more severe illness [10,11].

In the present study, we investigate the epidemiologic and viral molecular features of HAdVs circulating in Senegal after 4 consecutive years of sentinel surveillance of influenza-like Illness cases.

## Materiel and methods

### Samples and data collection

From January 2012 to December 2015 we collected specimens (nasal-pharyngeal and oral-pharyngeal swabs) and surveillance data for influenza and other viral respiratory pathogens from outpatients presenting with influenza-like-illness (ILI) at different sentinel sites in Senegal. Once collected, swabs are placed in 2-mL cryovials with viral transport medium (Universal Transport Medium; COPAN Diagnostics Inc., Murrieta, CA), and transported at a controlled temperature of 2°C—8°C to the laboratory. An ILI patient was defined as a person presenting with sudden onset of fever (>38°C) or history of sudden onset of fever in the recent past (≤ 3 days) and either cough or sore throat and/or rhinorrhea in the absence of other diagnosis, according to the CDC case definition. Each sample is accompanied by a case report form collecting demographic and clinical data. The questions included information on date of enrollment and symptom onset, sex, age, clinical symptoms, previous treatments, travelling history, vaccination status for influenza, and whether or not the patient was hospitalized. Upon arrival at the laboratory, the specimens were processed immediately for virus diagnosis. Aliquots of samples were also stored at −80°C for additional analysis (isolation and/or molecular characterization).

The data obtained daily were entered into an Epi Info database (Centers for Disease Control and Prevention, Atlanta, GA) and analyzed using Epi Info.

### Nucleic acid extraction and viral detection

Total viral nucleic acid (DNA and RNA) was extracted from 140 μl of each clinical specimen using the PureLink^™^ Viral RNA/DNA Mini Kit (Invitrogen, Carlsbad CA, USA) according to the manufacturer’s recommendation. DNA/RNA are eluted with 60 μl nuclease-free water and stored at −80°C until use.

A two-step multiplex real-time RT-PCR was performed with a Bio-Rad CFX-96 thermocycler (Bio-Rad Laboratories) and the Anyplex^™^ II RV16 Detection kit (Seegene) for a simultaneous testing of Influenza viruses (fluA and fluB), Human respiratory syncytial virus (RSVA and RSVB), Human adenoviruses (HAdV), Human metapneumovirus (HMPV), Human coronavirus (229E, NL63, OC43), Human parainfluenza virus (PIV1, -2, -3 and -4), Human rhinovirus (HRV), Human enterovirus (HEV) and Human bocavirus (HBoV), as previously described [12].

### Molecular characterization of respiratory adenoviruses strains between 2012 and 2015 in Senegal

In consideration with low Ct-values, 80 HAdV positives samples (20 per year) were selected using a random number generator on MS Excel for further molecular characterization using classical PCR and sequencing. Viral DNA was extracted as previously described and eluted with 50 μl water nuclease-free. DNAs were stored at −20°C until PCR reactions. For HAdV molecular characterization the last 300 base pairs (bp) of the hexon gene were amplified with the following specific primers: Adeno3 (5’-CCTTTGGCGCATCCCATTCT-3’) and Adeno4 (5’-TGGGCACCTATGACAAGCGC-3’) previously used by Garcia et al, [13]. The Phusion High-Fidelity PCR Master Mix with HF Buffer (New England Biolabs, Ipswich MA, USA) was used for amplifications. For each sample, PCR was carried out in a total reaction volume of 50 μl consisting of 15 μl H2O RNase free, 2.5 μl of each primer (diluted at 10μM), 25 μl of 2X Phusion Master Mix and 5 μl of DNA template. Cycling conditions were as follows: denaturation step of 15 min at 95°C, 40 PCR cycles including 30 s at 95°C, 60 s at 55°C, 60 s at 72°C followed by an extension step of 10 min of 72°C.

Five microliters of the PCR product was then mixed with 1 μl of 10X 5PRIME loading dye and loaded on to a 1% agarose gel along with an appropriated molecular weight markers (100 bp ladder, New England Biolabs), and gels were stained with ethidium bromide (0.5 μg/ml) before visualization under UV.

For positive samples (380 bp size band), amplicons were cut and purified using the GeneJET Gel Extraction Kit (Thermo Scientific). Purified products are then sent for sequencing to Beckman Coulter Services. Sequencing was performed in both directions with the same PCR primers (Adeno3 and Adeno4) on an ABI PRISM BigDye Terminator v3.1 Ready Reaction Cycle Sequencing kit (Applied Biosystems) on a 96-capillary ABI PRISM 3730-XL (Applied Biosystems). Data in FASTA format were then sent to the laboratory for analysis.

### Sequence analysis and multiple sequence comparison

Sequences successfully obtained were aligned with representative GenBank sequences of previously published genotypes using the BioEdit Sequence alignment Editor [14**]**. The search for sequence similarities were carried out using the Basic Local Alignment Search Tool (Blastn) from NCBI BLAST web portal. Phylogenetic trees were performed in MEGA 6 software [15] using the neighbor-joining method, and the statistical significance of the tree topology tested by bootstrapping (1,000 replicates). The evolutionary distances were derived using the Tamura-Nei method. Bootstrap replicates with values ≥70 are shown on the trees.

#### Statistical analysis

Regarding HAdV infection comparisons between age groups were performed using the Fisher’s exact test. P value < 0.05 was considered statistically significant and the 0–5 year age group was used as reference group. HAdV mono-infections were also compared to HAdV co-infections. The R.3.0.1 tool was used to perform the analyses.

### Ethical considerations

This study is a component of the 4S network syndromic surveillance [12]. The principles of the 4S network were approved by the Ministry of Health in its guidelines for influenza surveillance policy, finalized with the support of Pasteur Institute in Dakar and the Strengthening Influenza Sentinel Surveillance in Africa (SISA) project funded by the WHO. The protocol and oral consent were determined as routine surveillance activity, and therefore non-research by the Senegalese National Ethics committee and the steering committee for 4S network, an entity representing MoH, IPD, WHO and Clinicians in compliance with all applicable National regulations governing the protection of human subjects. Data were collected in an objective of surveillance and are anonymous. The information provided to participants was an informal description of the study. Respiratory specimens were collected, only after informed consent was granted, verbally, to local health care workers by the patients or parents in the case of minors. Oral consent was documented in the patient form with two questions about received information and about oral consent. Patients could refuse to participate, no specimen will be taken. For the surveillance activities, written consent is judged not necessary by the Senegalese national ethics committee, which has also previously approved the work of the National Influenza Center. Collections of non-sensitive data or an observation from normal care in which participants remain anonymous do not require ethics committee review. The patients included in this study were of all ages and consulted the sentinel sites due to influenza-like symptoms; the patients, or parents in the case of minors, accept the tests for respiratory viruses largely because they are free and safe.

## Results

### Patients’ characteristics

Between January 2012 to December of 2015 a total of 6381 samples were collected from patients meeting case definition for ILI at the different sentinel sites, and analyzed: 1213 (19%) from 2012, 1519 (23.8%) from 2013, 1930 (30.2%) from 2014 and 1719 (26.9%) during 2015 (Table 1). Patient ages ranged from 1 month to 95 years. The mean age was 10 years 11 months and median age was 4 years. Approximately the male/female ratio was 0.99 (3163 [49.6%] males and 3185 [49.9%] females). For 33 (0.52%) patients the sex was not documented. More than half of patients (51.7%; 3297/6381) were children of ≤ 5 years old followed by 5–10 years age group with 11.5% (731/6381) and 25–50 years age group with 10.9% (696/6381). Patients above 50 years old represented only 3.3% (210/6381) of enrolled patients and for 8.2% (526/6381) ages were not reported.

**Table 1:** Demographical characteristics of patients and most prevalent symptoms.

Characteristics	Years	2012	2013	2014	2015	Total
	Total Number	(N = 1213)	(N = 1519)	(N = 1930)	(N = 1719)	(N = 6381)
	**Gender** no. (%)					
	Female	587(48.39)	767(50.49)	987(51.14)	844(49.10)	**3185(49.9)**
	Male	610(50.29)	744(48.98)	936(48.50)	873(50.79)	**3163(49.6)**
	Missing	16(1.32)	8(0.53)	7(0.36)	2(0.12)	**33(0.52)**
	**Age** no. (%)					
	0–5 yrs	749(61.75)	758(49.90)	941(48.78)	849(49.39)	**3297(51.7)**
	5–10 yrs	117(9.65)	163(10.73)	208(10.78)	243(14.14)	**731(11.5)**
	10–15 yrs	56(4.62)	72(4.74)	99(5.13)	109(6.34)	**336(5.3)**
	15–25 yrs	78(6.43)	125(8.23)	227(11.76)	155(9.02)	**585(9.2)**
	25–50 yrs	62(5.11)	124(8.16)	282(14.61)	228(13.26)	**696(10.9)**
	50+ yrs	21(1.73)	23(1.51)	91(4.72)	75(4.36)	**210(3.3)**
	Missing	130(10.72)	254(16.72)	82(4.25)	60(4.49)	**526(8.2)**
	**Clinical signs** no. (%)					
	Myalgia	125(10.31)	327(21.53)	306(15.85)	302(17.57)	**1060(16.6)**
	Fever	1129(93.08)	1350(88.87)	1864(96.58)	1652(96.10)	**5995(93.9)**
	Cough	824(67.93)	1099(72.35)	1554(80.52)	1373(79.87)	**4850(76.0)**
	Vomiting	124(10.22)	33(2.17)	94(4.87)	148(8.61)	**399(6.2)**
	Diarrhea	94(7.75)	30(1.97)	43(2.23)	39(2.27)	**206(3.2)**
	Headache	105(8.66)	182(11.98)	263(13.63)	305(17.74)	**855(13.4)**
	Dyspnea	20(1.65)	24(1.58)	87(4.51)	24(1.40)	**155(2.4)**
	Rhinitis	734(60.51)	959(63.13)	741(38.39)	522(30.37)	**2956(46.3)**
	Pharyngitis	109(8.99)	151(9.94)	409(21.19)	318(18.50)	**987(15.5)**

Among patients the most common respiratory symptoms were fever (94%; 5995/6381), cough (76%; 4850/6381) and rhinitis (46.3%; 2956/6381). Myalgia, pharyngitis, headache, dyspnea and diarrhea were also reported in smaller proportions.

### Patients and adenoviral infection

Of 6381 specimens tested, 1967 (30.8%) were positive for HAdV (Table 2). Detection rates over the study period are almost similar in the first 3 years (2012, 2013 and 2014) while in 2015 there is a marked decrease in adenoviral infections. The mean age of infected patients was 8 years 7 months and median age was 3 years.

**Table 2:** Detection rates of human adenovirus infection in patients with ILI per year from 2012 to 2015 in Senegal and comparison of the distribution into the different age groups.

Year	2012	2013	2014	2015	Total	
Total Number	(N = 1213)	(N = 1519)	(N = 1930)	(N = 1719)	(N = 6381)	P-values
Positivity (per year)	34.71	33.2	33.2	23.3	30.8	
Sex male infection	224(53.2)	252(49.9)	313(48.8)	204(51.0)	993(50.5)	0.373
**Age and infection**						
0_5 years	324(80.0)	283(56.0)	372(58.0)	245(61.2)	1224(62.2)	< 2.2e-16
5_10 years	30(7.1)	47(9.3)	59(9.2)	54(13.5)	190(9.7)	
10_15 years	16(3.8)	20(4.0)	27(4.2)	15(3.7)	78(4.0)	
15_25 years	15(3.6)	32(6.3)	64(10.0)	22(5.5)	133(6.8)	
25_50 years	11(2.6)	35(6.9)	68(10.6)	38(9.5)	152(7.7)	
50+ years	5(1.2)	9(1.8)	26(4.1)	14(3.5)	54(2.7)	
Missing	20(4.7)	79(15.6)	25(3.9)	12(3.0)	136(6.9)	
Total	421(34.7)	505(33.2)	641(33.2)	400(23.3)	1967(30.8)	

From the 1967 adenovirus positive cases, 1561 (79.4%) were found in co-infection with at least one respiratory virus. The most common were influenza viruses (53.1%; 1045/1967), rhinoviruses (30%; 591/1967), enteroviruses (18.5%; 364/1967) and RSV (13.5%; 266/1967).

Regarding the viral detection per age group, most of HAdV infected cases (62.2%; 1224/1967) were under 5 years patients, a statistically significant finding (p <0.05). However, the detection rates in the other groups including the elderly (above 50 years old) remain high. No significantly gender distribution of adenoviral infection was observed.

The comparison of symptoms prevalence between ILI patients with adenoviral infection and patients without adenoviral infection showed that cases of myalgia (P = 0.0014), cough (P = 0.0028), diarrhea (P < 0.001), rhinitis (P < 0.001) and headache (P = 0.01) are significantly higher in patients infected by adenoviruses (Table 3).

**Table 3:** Adenoviral infection and clinical signs in patients with ILI in Senegal, 2012–2015.

Year	2012	2013	2014	2015	Total	P-values
Total Number	(N = 1213)	(N = 1519)	(N = 1930)	(N = 1719)	(N = 6381)	
Positivity (per year)	34.71	33.25	33.21	23.27	30.83	
**Clinical signs** no. (%)						
Myalgia	29(6.9)	116(23.0)	78(12.2)	60(15.0)	283(14.4)	**0.001437**
Fever	396(94.1)	459(90.9)	613(95.6)	383(95.7)	1851(94.1)	0.9138
Cough	291(69.1)	386(76.4)	534(83.3)	331(82.7)	1542(78.4)	**0.002881**
Vomiting	55(13.1)	7(1.4)	30(4.7)	43(10.7)	135(6.9)	0.1789
Diarrhea	42(10.0)	14(2.8)	19(3.0)	14(3.5)	89(4.5)	**9.194e-05**
Headache	27(6.4)	67(13.3)	72(11.2)	68(17.0)	234(11.9)	**0.01864**
Dyspnea	5(1.2)	4(0.8)	29(4.5)	2(0.5)	40(2.0)	0.1707
Rhinitis	278(66.0)	344(68.1)	253(39.5)	106(26.5)	981(49.9)	**0.0001482**
Pharyngitis	29(6.9)	45(8.9)	118(18.4)	60(15.0)	252(12.8)	**8.95e-05**

Values in bold are considered statistically significant

**Fig 1:**
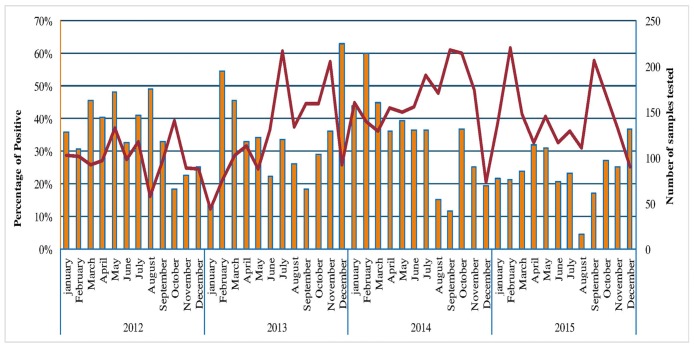
Distribution of adenovirus-positive cases among patients with influenza-like illness, by month and year. The curve represents the total number of influenza-like Illness cases tested for adenovirus. Bars represent the proportions (rates) of adenovirus-positive cases for each month.

The Fig 1 shows the temporal distribution of HAdV positivity rate per month in Senegal from 2012 to 2015. We noted that HAdV was detected throughout the year at a high level with detection peaks of different amplitude. The highest peak, with 62% of detection rate, was recorded on December 2013. HAdV circulation pattern shows no seasonality even if results suggest a higher activity of these viruses during cold periods. It should be pointed out that the cold periods (between December and February) experience some instability in Senegal with possibilities of shifting.

### Phylogenetic analysis and typing of HAdV

For phylogenetic analysis, we were able to obtain the partial hexon gene sequence from 54 HAdV-positive samples: 8 were from samples in 2012, 13 from 2013, 11 from 2014, 16 from 2015 and 6 from 2016. Unfortunately, some samples showed no amplification or poor-quality sequences. The low sensitivity of conventional PCR compared with real time PCR on samples with low viral load, and certainly non-specific amplifications could be the cause of these failures.

**Fig 2:**
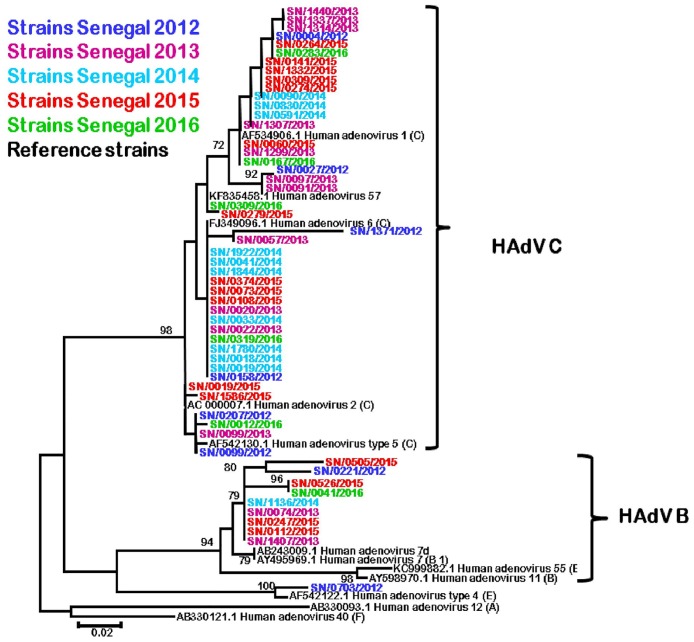
Molecular typing of Human Adenoviruses (HAdV) detected in patients with ILI in Senegal from 2012 to 2016. The last 300 nucleotides of the adenovirus hexon gene were amplified, sequenced, and compared to published sequences (in black) from GenBank using the neighbor-joining method with 1000 bootstrap replicates with MEGA 6 version. Senegalese strains are represented in different colors depending on the year of detection.

The nucleotide sequence alignment clustered the majority of Senegalese isolates into HAdV-C species (44/54). 9 isolates grouped with HAdV-B species and the remaining isolate, from 2012, seems close to the HAdV- 4 genome type belonging to the HAdV-E species. In all cases bootstrap values are high (more than 85%). Within the HAdV-C species, 16 Senegalese isolates are grouped with the type HAdV-6 (36.4%); 2 isolates with HAdV-2 type (4.5%), 4 with HAdV-5 type (9%), and 22 isolates formed a subcluster with HAdV-1 and 57 types (Fig 2). The 9 HAdV-B like species from Senegal grouped with genome types HAdV-7, HAdV-55 and HAdV-11 as shown by a phylogenetic branch with a high bootstrap value of (88%). We also noted that this dominance of species C and B is confirmed over the years.

## Discussion

In this four-year retrospective study, we characterized HAdV isolates derived from an ILI surveillance program conducted as collaboration between Pasteur Institute of Dakar and the Senegalese Ministry of Health between 2012 and 2015. It is the first nationally molecular epidemiology investigation of HAdVs and even in West Africa.

Our study suggests that HAdV are strongly associated with ILI syndrome in Senegal with an overall detection rate of 30.8% among 6381 patients. This rate seems very higher in comparison with available data. Indeed, much lower rates are reported in similar other studies conducted in other countries. A study conducted in Kenya [16] on refugees from different countries (Somalia, Sudan, Ethiopia and Kenya) yielded a detection rate of 21.7%, in Gabon Douki et al [17] detected HAdV in 16.3% of outpatients with ILI. These detection rates are still lower in other geographical regions: South Korea with 10.1% or 0.6% [18,19], China with 2.7% or 6.3% [20,21], Philippines with 0.9% [22], Malaysia less than 2% [23], USA with 5.7% or 2.8% reported [24,25], Canada in the Ontario Provence with 0.9% [26], Peru with 6.2% [27], Venezuela with 1.6% [28], England with 6.6% [29]. The analysis of these data tends to confirm a higher prevalence of adenoviruses in the respiratory sphere in African populations. This trend was largely confirmed when we investigated the importance of HAdV in children with acute respiratory infections. Indeed we observed that proportions in Cameroon (27.3%) [30] and Senegal (29.2%) (Fall et al, on submission) were considerably higher than those found in other geographical areas: Nascimento-Carvalho et al., [31] in Brazil with 3%, Moe et al., [32] in Norway (1.7%), Wansaula et al., [33] in USA (1%) or Lu et al., [34] in China.

However, these discrepancies in HAdV detection rates can be also due to differences in technical approaches, virus burden geographical differences, the number of patients tested, the periods during which samples were collected and even the duration of the study. It should be also noted that adenoviral detection does not necessarily prove disease causation as coincidental upper airway infection, asymptomatic viral carrier state [35], or prolonged shedding [36] in a previous infection could explain adenoviral detection.

Regarding the group age, as expected, results showed that most patients with HAdV infection were younger than 5 years (62.2%), a statistically significant finding. These results are in concordance with those of other studies which findings concluded that most children are infected by adenovirus at an early age [25,37,38–41]. Indeed, it is well established that by 5 years of age, 70% to 80% of children demonstrate antibodies to at least one serotype [42]. Additionally more than 80% of diagnosed HAdV infections occur in children < 4 years old (due to lack of humoral immunity) [43]. Although most cases exhibit low to mild symptoms are and indistinguishable from other viral causes, acute respiratory infections caused by HAdV can be severe [44], or even fatal [7,45], and are associated with the highest risk of long term respiratory sequelae [46].

Consistent with the report from many other studies, results here showed that 79.4% of HAdV infected participants were co-infected with one or more other respiratory tract viruses. The most frequently co-detected viruses were influenza viruses (53.1%), rhinoviruses (30%), enteroviruses (18.5%) and RSV (13.5%). However, we noted no significant differences in clinical characteristics and laboratory findings between patients with single HAdV infection and those co-infected. The same observation was reported in studies conducted in diverse geographical contexts [27,41]. A previous study conducted in Chilean children stated that the clinical severity in patients with single HAdV infection and those with mixed infections was the same [47]. The overall finding is that the clinical value of such co-infections is not clear and still requires independent investigations in order to assess the association between co-infection and severe illness or symptoms.

Regarding the four years of surveillance, HAdV circulation pattern shows no clear seasonality even if results suggest a higher activity of these viruses during cold periods. This lack of seasonality of HAdV infection has been largely reported elsewhere [27,48,49]. However, seasonal peaks for HAdV infection were noted in summer in some China areas [50] or in spring in Northern China [51], Mexico [52] and Taiwan [53].

In our study, the last 300 bp region of the hexon gene were used for molecular studies of the different HAdV isolates. Phylogenetic analysis showed that among the 54 sequenced strains HAdV-C species were the most common HAdV detected (81.5%) in patients with ILI in Senegal from 2012 to 2016. Despite some divergences, the strains from Senegal were close to types 1, 2, 5, 6 and 57. This HAdV-C species predominance was reported in Malaysia [23], in Italy [54], in many Latina America countries [27,52,55] in contrast with studies done in the United States of America [56], United Kingdom [37], Korea [57], in Argentina [58] and China [59], where HAdV-B species were the most commonly isolated HAdV.

HAdV-B species were the second most common in Senegal with 9 strains, and only one type belonging to HAdV-E species was sequenced. HAdV-B species from Senegal clustered with genome types HAdV-7, HAdV-7d, HAdV-55 and HAdV-11 (88% bootstrap value). HAdV-7d serotype, firstly identified in 1980 in Beijing [59], is of particular concern as it was often associated with illnesses presenting with more severe and higher levels of morbidity than other respiratory HAdV pathogens, and also may result in higher levels of fatalities [60–62]. The HAdV-55 genome type, formerly known as HAdV-11a, is a genotype resulting from recombination between HAdV-11 and HAdV-14 [63]. The serotype has recently reemerged as a highly virulent pathogen, causing severe [64] and sometimes fatal pneumonia among immunocompetent adults, particularly in Asia [65–67]. So the circulation of such HAdV genome types in Senegal emphasizes the need to reinforce HAdV surveillance, especially in hospitalized patients, by including HAdV genome detection and genotyping in the documentation of severe respiratory infections.

The single HAdV-E species strain was typed as HAdV-4, the unique human type in this species, which is more commonly associated with high rates of febrile respiratory illness in US military recruits [68] though associated with viral conjunctivitis outbreak in Australia [69] for example.

We observed some limitations in our study. First, considering the vast number of HAdV positive samples, only a small number of HAdV were typed. So the sequencing results do not reflect the full spectrum of HAdV strains that may be circulating in ILI patients in Senegal, and even for selected samples it may have a bias toward samples with a high viral load. Another limitation concerned the molecular methods used for typing HAdVs in this study, a method which targeted a short hexon hypervariable region that has been shown to correlate closely with serotype. This method does not provide genomic detail and might miss recombination events located in other regions of the genome. Therefore, full-genome sequencing would be more informative on Senegalese strains, especially for HAdV-B7 and HAdV-B55 types. The results of this study should also be interpreted with caution especially for HAdV ILI causality (carriage in healthy or asymptomatic individuals).

## Conclusion

In conclusion, the results of the present study suggest strong year-round HAdV activity in Senegal, especially in children up to 5 years of age. Molecular studies revealed that the dominant species in circulation in patients with ILI appears to be HAdV-C, HAdV-B species. The circulation of though HAdV-7d and HAdV-55 genome types is of note as these serotypes are recognized causes of more severe and even fatal acute respiratory infections. So in the interest of global public health we strongly suggest molecular surveillance and genotyping of newly detected HAdV strains in Senegal and even by whole genome sequencing for some especial strains. Our study offers also an important perspective on the burden of adenovirus-associated respiratory illness in Senegal. Such a perspective, especially among children, should include asymptomatic controls, SARI cases, information on disease outcome, atypical clinical signs, duration of symptoms, and treatment. Data regarding viral load, shedding, and other possible etiologies (e.g., bacterial and other viruses) would also enable a more thorough assessment of the viral effective disease (or symptom) causality.
